# Differentiation signals induce *APOBEC3A* expression via GRHL3 in squamous epithelia and squamous cell carcinoma

**DOI:** 10.21203/rs.3.rs-3997426/v1

**Published:** 2024-03-04

**Authors:** Nicola J. Smith, Ian Reddin, Paige Policelli, Sunwoo Oh, Nur Zainal, Emma Howes, Benjamin Jenkins, Ian Tracy, Mark Edmond, Benjamin Sharpe, Damian Amendra, Ke Zheng, Nagayasu Egawa, John Doorbar, Anjali Rao, Sangeetha Mahadevan, Michael A. Carpenter, Reuben S. Harris, Simak Ali, Christopher Hanley, Rémi Buisson, Emma King, Gareth J. Thomas, Tim R. Fenton

**Affiliations:** 1 School of Cancer Sciences, Faculty of Medicine, University of Southampton, UK; 2 School of Biosciences, University of Kent, UK; 3 Bio-R Bioinformatics Research Facility, Faculty of Medicine, University of Southampton, UK; 4 Department of Biological Chemistry, School of Medicine, University of California Irvine, Irvine, CA, USA; 5 Department of Pathology, University of Cambridge, UK; 6 Gilead Sciences, Research Department, 324 Lakeside Dr. Foster City, CA 94404, USA; 7 Department of Biochemistry and Structural Biology, University of Texas Health San Antonio, San Antonio, TX 78229, USA; 8 Howard Hughes Medical Institute, University of Texas Health San Antonio, San Antonio, TX 78229, USA; 9 Department of Surgery and Cancer, Imperial College London, Hammersmith Hospital Campus, London, UK.; 10 Institute for Life Sciences, University of Southampton, UK

**Keywords:** APOBEC3A, cancer mutagenesis, GRHL3, keratinocyte, differentiation, HNSCC

## Abstract

Two APOBEC (apolipoprotein-B mRNA editing enzyme catalytic polypeptide-like) DNA cytosine deaminase enzymes (APOBEC3A and APOBEC3B) generate somatic mutations in cancer, driving tumour development and drug resistance. Here we used single cell RNA sequencing to study *APOBEC3A* and *APOBEC3B* expression in healthy and malignant mucosal epithelia, validating key observations with immunohistochemistry, spatial transcriptomics and functional experiments. Whereas *APOBEC3B* is expressed in keratinocytes entering mitosis, we show that *APOBEC3A* expression is confined largely to terminally differentiating cells and requires Grainyhead-like transcription factor 3 (GRHL3). Thus, in normal tissue, neither deaminase appears to be expressed at high levels during DNA replication, the cell cycle stage associated with APOBEC-mediated mutagenesis. In contrast, we show that in squamous cell carcinoma tissues, there is expansion of *GRHL3* expression and activity to a subset of cells undergoing DNA replication and concomitant extension of *APOBEC3A* expression to proliferating cells. These findings indicate a mechanism for acquisition of APOBEC3A mutagenic activity in tumours.

## Introduction

The *APOBEC3A* and *APOBEC3B* (apolipoprotein B mRNA editing catalytic polypeptide-like 3A and 3B) genes encode two closely related DNA cytosine deaminases that belong to the seven-protein human APOBEC3 family. The APOBEC3 enzymes convert deoxycytidine to deoxyuridine in single-stranded DNA (ssDNA), a mutagenic activity that explains at least in part their ability to restrict replication of retroviruses and endogenous retroelements through targeting nascent cDNA during reverse transcription^1,2^. In addition, APOBEC3A and APOBEC3B have evolved functions in the cell nucleus including transcriptional regulation^3,4^ and responses to nuclear-resident viruses^5–10^. Acquisition of these nuclear functions appears to have come at a cost however, as both APOBEC3A and APOBEC3B have been implicated in generating somatic mutations (mainly C>T transitions and C>G tranversions at TpC sites) in cancer cell genomes, driving cancer development and therapeutic resistance^11–22^. Two mutation signatures attributed to APOBEC3A/B activity have been observed in multiple cancer types but although extensive biochemical and genetic data support the involvement of both APOBEC3A and APOBEC3B in somatic mutagenesis, their gene expression levels as determined by analysis of bulk tumour data or in cancer cell lines that display the signatures are only weakly, if at all, correlated with the presence of these mutation signatures^23–31^. Accurately determining the conditions under which *APOBEC3A* and *APOBEC3B* are expressed in normal and cancerous tissues represents a key challenge in building our understanding of how APOBEC-mediated mutagenesis occurs, and how they might be targeted for cancer treatment^32^. This objective is complicated however, by their expression in immune cells, which are frequently present at high levels in tumour biopsies, and until recently by a lack of specific antibodies for *in situ* analysis. Here, we addressed these challenges by conducting single cell RNA sequencing (scRNA-seq) of matched normal and tumour samples from patients with head and neck squamous cell carcinoma (HNSCC), a tumour type in which high burdens of APOBEC signature mutations are frequently observed, with evidence pointing to roles for both APOBEC3A and APOBEC3B in generating these mutations^23–25,33–35^. We analysed *APOBEC3A* and *APOBEC3B* gene expression patterns in these data and in additional published scRNA-seq datasets from healthy and cancerous epithelial tissues, deploying recently developed antibodies in immunohistochemical analysis of tissue sections to corroborate our findings at the protein level. We used computational methods to predict transcription factors responsible for regulating APOBEC expression and validated our predictions in near-normal immortalized keratinocytes (NIKS)^36^, identifying Grainyhead-like transcription factor 3 (GRHL3) as a novel regulator of *APOBEC3A* expression in terminally differentiating keratinocytes. In contrast, and consistent with findings from different cell types^37,38^, *APOBEC3B* expression is confined to proliferating cells, with highest levels evident in G2/M-phase of the cell cycle. In HNSCC, we find evidence of GRHL3 activity and *APOBEC3A* in a subpopulation of tumour cells undergoing DNA replication; the context in which mutagenic APOBEC activity is postulated to occur due to deamination of lagging strand ssDNA exposed at the replication fork^39–44^. Our findings provide new insight into the transcriptional control of APOBEC3A gene expression in squamous epithelia and provide a potential mechanism for the acquisition of APOBEC3A-induced mutations in cancer.

## Results

### *APOBEC3A* is expressed in epithelial cells from healthy tonsil and oesophagus.

**Figure 1:**
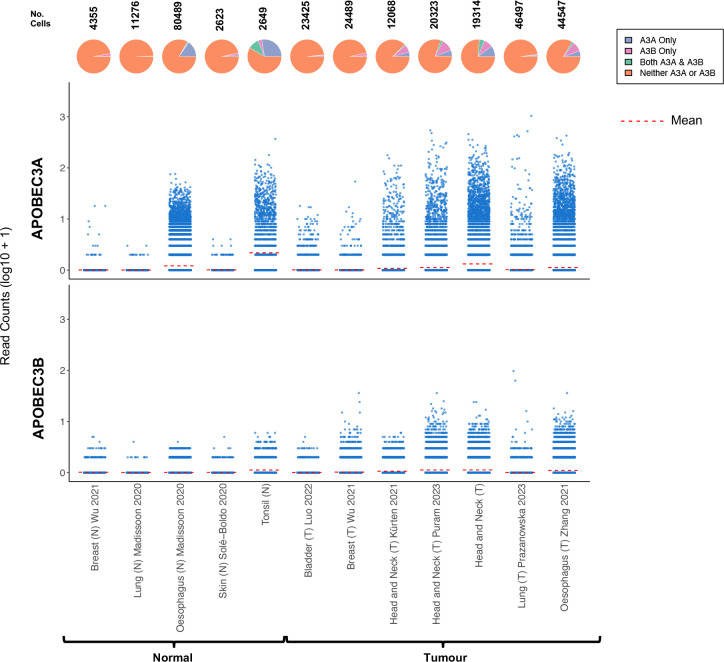
*APOBEC3A* and *APOBEC3B* expression in scRNA-seq datasets representing normal and tumour epithelial cells from tissues in which cancers that display prominent APOBEC mutational signatures arise. The number above each pie chart represents the total number of epithelial cells in each dataset. The references for each dataset are provided in Supp Table 2.

Although most cancers that display enrichment for APOBEC mutational signatures are carcinomas, i.e. tumours that arise from epithelial cells, little is known about the expression patterns and physiological regulation of *APOBEC3A* and *APOBEC3B* in healthy epithelium, or regarding the proportion of malignant cells that express *APOBEC3A*, *APOBEC3B* or both genes in tumour biopsies. To address these knowledge gaps, we assembled scRNA-seq data from the epithelial cells (see Methods) present in 10 oropharyngeal SCC samples and 7 matched normal (contralateral tonsil) samples from patients undergoing surgical resection at our institution (Table S1), together with 11 published scRNA-seq datasets from healthy skin, breast and oesophagus and from cancers of the breast, bladder, head and neck (HNSCC), oesophagus (ESCC) and lung (all cancers that typically display moderate to strong enrichment for APOBEC mutation signatures, Table S2). Very few *APOBEC3A* or *APOBEC3B* expressing epithelial cells were present in the normal skin, breast or lung datasets but 16.4% of epithelial cells from normal oesophagus and 38.1% from normal tonsil expressed *APOBEC3A*, 10.5% of which also expressed *APOBEC3B* (Figure 1). 3.5% of tonsil epithelial cells expressed only *APOBEC3B* (Figure 1). In the tumour samples, the majority of epithelial (tumour) cells expressed neither *APOBEC3A* nor *APOBEC3B* at levels detectable by scRNA-seq, and the only datasets containing a significant number of cells expressing *APOBEC3A* and/or *APOBEC3B* were from HNSCC or ESCC (Figure 1). Only the datasets from healthy tonsil and oesophagus and from HNSCC and ESCC contained sufficient *APOBEC3A* and/or *APOBEC3B* expressing cells to permit further analysis, so we initially interrogated the data from tonsil epithelial cells, the dataset in which we observed the highest average *APOBEC3A* expression per cell and the greatest proportion of *APOBEC3A*- and/or *APOBEC3B*-positive cells (Figure 1).

### APOBEC3A and APOBEC3B are expressed in distinct cell sub-populations in healthy tonsil epithelium.

**Figure 2:**
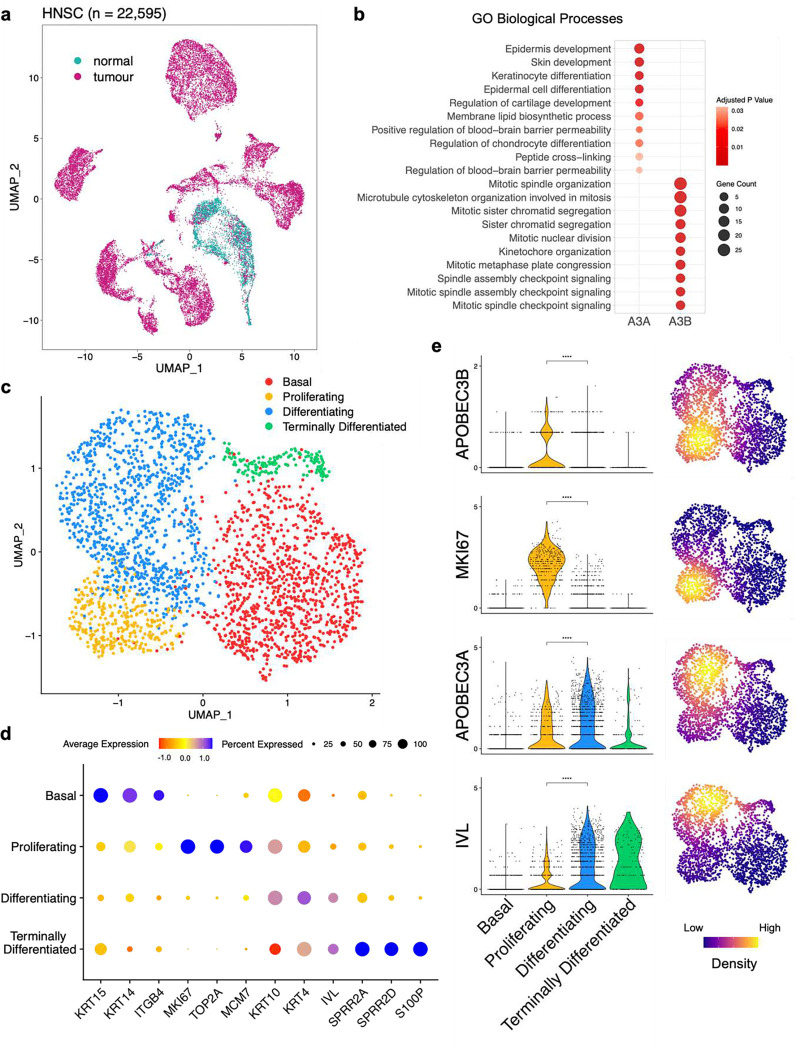
*APOBEC3A* and *APOBEC3B* are expressed in different subsets of tonsillar epithelial cells **a)** UMAP projection of epithelial cells from oropharyngeal squamous cell carcinoma samples (n = 10), and matched normal tonsil (n = 7). **b)** Pathway analysis of genes that were the most co-expressed with APOBEC3A and APOBEC3B. **c)** UMAP projection depicting four phenotypes (basal, proliferating, differentiating, terminally differentiated) displayed by the normal tonsillar epithelial cells in our dataset. **d)** Marker genes used to identify the four epithelial phenotypes represented in panel c. **e)** Violin plots of gene expression in individual tonsillar epithelial cells, and UMAP projections of the density of gene expression in the tonsillar epithelial subtypes. (**** = p-value < 0.0001, Wilcoxon’s Rank Sum Test).

Since most *APOBEC3A*-expressing cells in the normal tonsil dataset did not co-express detectable levels of *APOBEC3B*, we were interested to determine whether the cells comprising this *APOBEC3A*-positive / *APOBEC3B*-negative population might share a common phenotype and if so, whether it might be distinct from the *APOBEC3A*-negative / *APOBEC3B*-positive, *APOBEC3A/B*-positive and *APOBEC3A/B*-negative populations. To address this question, the 2,649 epithelial cells derived from normal tonsil were subset from our 21,937 epithelial cell dataset (Figure 2a) and pathway analysis using gene ontology biological processes (GOBP) was performed on the top 100 genes co-expressed with either *APOBEC3A* or *APOBEC3B* (Table S3). Considering only the top ten GOBP pathway hits for the two APOBECs, there was no overlap, and each was dominated by different biological processes. For *APOBEC3A* the top ten pathways included those involved in epidermal and keratinocyte development and differentiation whereas, consistent with observations in bulk RNA-seq data from breast cancer^45^, all processes in the top ten for *APOBEC3B* were associated with mitosis (Figure 2b, Table S4). This finding suggested that in healthy tonsil epithelium, *APOBEC3B* is expressed in cycling cells undergoing cell division while *APOBEC3A* is restricted to those keratinocytes undergoing terminal differentiation. To further investigate this possibility, the epithelial cells were clustered based on known markers for different epithelial cell states^46–49^. These genes included markers for basal cells (cytokeratin-14 (*KRT14*) and cytokeratin-15 (*KRT15*), proliferating epithelium (Ki-67 (*MKI67*), minichromosome maintenance complex component 7 (*MCM7*)), differentiating keratinocytes (involucrin (*IVL*), cytokeratin-10 (*KRT10*)), and terminally differentiating keratinocytes (*SPRR2A*, *S100P*) (Figure 2c, d). Similarly, scRNA-seq of healthy oesophageal epithelium^50^ had previously been clustered into four distinct epithelial phenotypes: basal epithelium (‘epi-basal’), a proliferating suprabasal epithelium (‘epi-suprabasal’, differentiating stratified epithelium (‘epi-stratified’), and terminally differentiated upper epithelium (‘epi-upper’) (Figure S1a). Marker gene expression patterns for these epithelial subtypes resembled those observed in the corresponding tonsillar epithelial subtypes (Figure S1b).

As inferred from the pathway analysis, *APOBEC3B* was expressed predominantly in proliferating cells, significantly more so than in differentiating cells (p < 0.0001, Wilcoxon’s Rank Sum Test), exhibiting a similar expression profile to *MKI67* (Figure 2e). While also expressed in a subset of proliferating cells, *APOBEC3A* expression was significantly higher in differentiating cells (p < 0.0001, Wilcoxon’s Rank Sum Test), and was also detectable in some terminally differentiated cells following the expression pattern of *IVL* (Figure 2e). Although *APOBEC3A* was expressed in a lower proportion of healthy oesophageal epithelial cells compared to those of the tonsillar epithelium, it was again co-expressed with *IVL* in differentiating cells and expressed weakly if at all, in the proliferative compartment (p < 0.0001, Wilcoxon’s Rank Sum Test, Figure S1c).

### Keratinocyte cell cycle exit and initiation of differentiation is marked by a switch from *APOBEC3B* to *APOBEC3A* expression.

**Figure 3:**
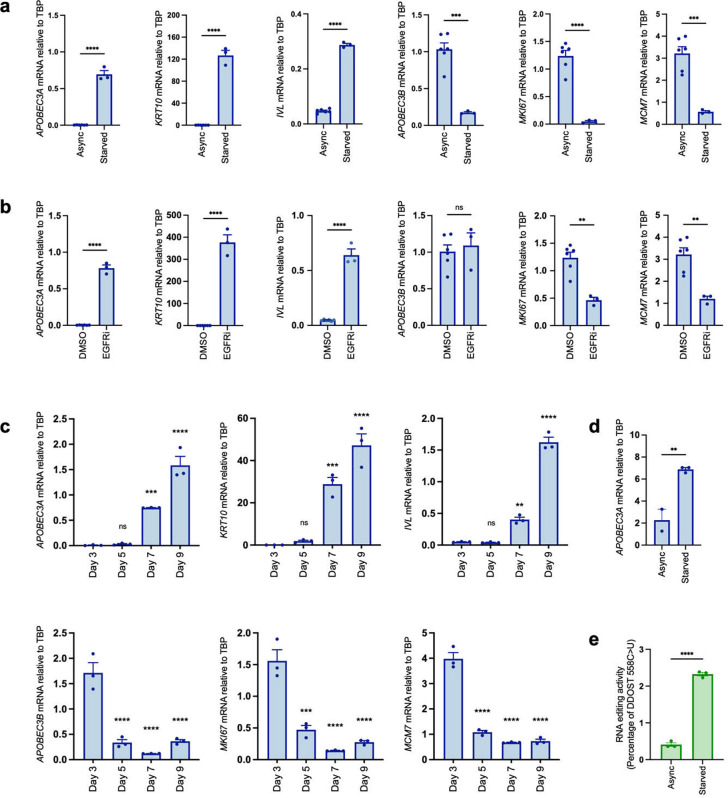
Keratinocyte cell cycle exit and initiation of differentiation is marked by a switch from *APOBEC3B* to *APOBEC3A* expression. qRT-PCR-based gene expression measurements for *APOBEC3A*, *KRT10*, *IVL, APOBEC3B*, *MKI67* and *MCM7* in: **(a)** proliferating NIKS (Async) or following 48 hours of growth factor deprivation (Starved); **(b)** NIKS following 24 hours of vehicle control (DMSO) or 100 nM afatinib treatment (EGFRi); **(c)** NIKS collected 3, 5, 7, or 9 days after plating. **d)** qRT-PCR measurements of *APOBEC3A* expression in primary human epidermal keratinocytes (NHEK) growing in full medium (Async) or following 48 hours of growth factor deprivation (Starved). **e)** Percentage of DDOST transcripts that were C>U edited at c558 in asynchronous growing NIKS (Async) and following 48 hours of growth factor withdrawal (starved) measured by digital PCR assay. All data derived from at least three independent experiments, error bars = SEM. * = p-value < 0.05; ** = p-value < 0.01; *** = p-value < 0.001; **** = p-value < 0.0001. Pairwise comparisons were performed using unpaired two-tailed t-tests in (a), (b), (d) and (e) and comparisons of mRNA levels on days 5, 7 and 9 to day 3 in (c) were performed using one-way ANOVA with Dunnett’s multiple comparisons test.

Our finding that *APOBEC3A* is expressed in differentiating epithelial cells of the tonsil and oesophagus is consistent with a previous report that it is upregulated during Ca^2+^-induced differentiation of W12 cells (a cell line established from a cervical neoplasia that harbours HPV16^51^) and normal human epidermal keratinocytes (NHEK)^52^. In the same study, *APOBEC3B* upregulation was also observed in W12 cells following 10 days in high Ca^2+^ but not in NHEKs, suggesting an HPV-specific induction, as reported^33,53,54^. Ca^2+^ is an activator of protein kinase C (PKC) signalling, which also mediates potent induction of *APOBEC3A* by phorbol esters in keratinocytes^55–57^. We therefore sought to uncouple potential PKC-dependent effects from differentiation-dependent effects on *APOBEC3A* expression by using three other established methods for inducing keratinocyte differentiation: growth to high density; inhibition of epidermal growth factor receptor (EGFR); and growth factor withdrawal^58,59^. In all three contexts we observed upregulation of *APOBEC3A*, along with *IVL* and *KRT10* and either no change (following 24 hours of the EGFR inhibitor afatinib) or a decrease (following growth to high density, serum and growth factor withdrawal) in *APOBEC3B* expression, closely mirroring decreases in *MKI67* and *MCM7* (Figure 3 a – c). In contrast to *APOBEC3A* induction by phorbol 12-myristate 13-acetate (PMA), the upregulation observed following growth factor withdrawal was independent of PKC signalling (Figure S2a) and was also observed in primary keratinocytes (Figure 3d). The increase in *APOBEC3A* mRNA was accompanied by an increase in C>U editing of the Dolichyl-Diphosphooligosaccharide--Protein Glycosyltransferase Non-Catalytic Subunit (DDOST) mRNA at c558, a specific readout for APOBEC3A deamination activity^30,60^ (Figure 3e). Upon treating NIKS with inhibitors of the two major mitogenic signalling pathways downstream of EGFR (RAS/MEK/ERK and PI3K/AKT/mTOR) we observed induction of *APOBEC3A* only by the MEK inhibitor trametinib, which was also the only compound to induce *IVL* / *KRT10* expression (Figure S2b, left panels) and to reduce *MKI67* and *MCM7* expression (Figure S2b, right panels). Interestingly the ERK1/2 inhibitor ravoxertinib did not block proliferation (as measured by *MKI67* and *MCM7* expression), nor did it induce *APOBEC3A* or differentiation markers. PI3K (pictilosib), AKT (MK2206) and mTORC1 (everolimus) inhibitors had no effect on proliferation or *APOBEC3A* expression but they did reduce *APOBEC3B* expression, which unlike *MKI67* and *MCM7*, was unaffected by MEK inhibition (Figure S2b, right panels). PI3K inhibition has previously been shown to reduce *APOBEC3B* expression in the U2OS human osteosarcoma cell line, via effects on NFκB and AP-1 activity^61^.

Taken together, our results from human tissue samples and the experiments in NIKS suggest that cell cycle exit and initiation of terminal differentiation in keratinocytes is accompanied by a switch in *APOBEC3* gene usage, from *APOBEC3B*, which is expressed in cycling cells entering cell division, to *APOBEC3A*.

### APOBEC3A expression is induced by Grainyhead-like transcription factor 3 during keratinocyte differentiation.

**Figure 4:**
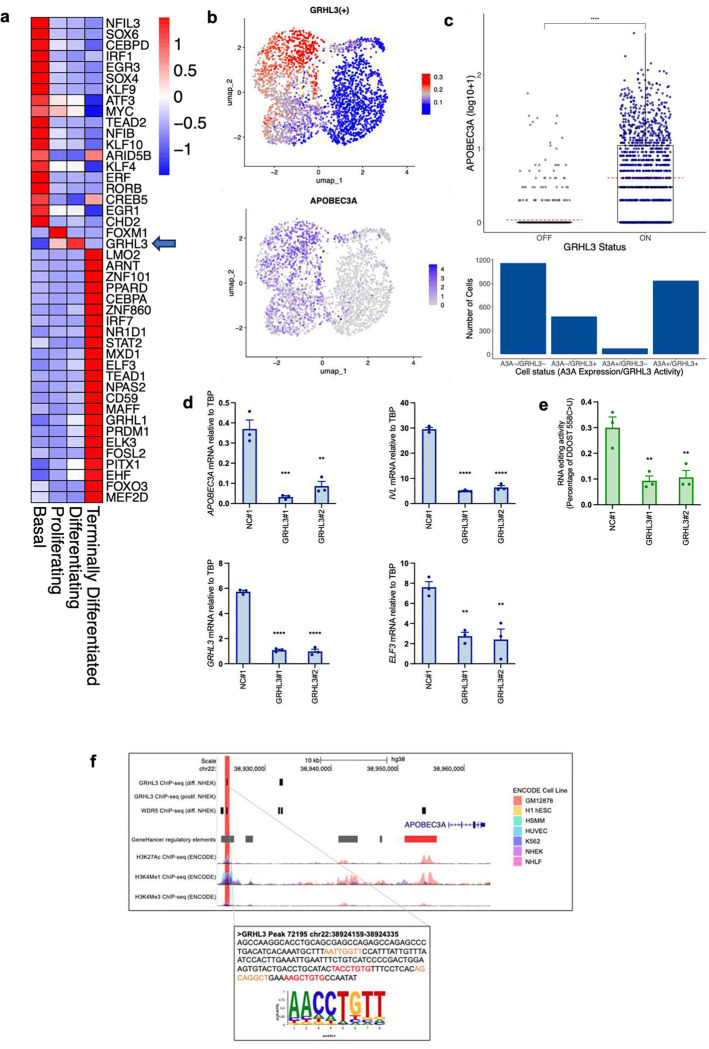
APOBE3A expression is induced by Grainyhead-like transcription factor 3 during keratinocyte differentiation. **a)** Heatmap showing those transcription factors (of the 363 with a SCENIC activity score in our scRNA-seq dataset from healthy tonsil epithelium) that were differentially expressed (fold change > 1.1, adjusted p-value <0.05) between the clusters defined in Figure 2c. **b)** UMAPs showing GRHL3 transcription factor activity score from SCENIC (top) and *APOBEC3A* expression (bottom) in the Southampton scRNA-seq dataset from healthy tonsil epithelium. **c)** boxplot showing *APOBEC3A* expression stratified by SCENIC binary predictions of GRHL3 ‘off’ or GRHL3 ‘on’ (top; (**** = p-value < 0.0001, Wilcoxon’s Rank Sum Test)) and histogram showing the number of cells in each of four groups: GRHL3 ‘off’, no detectable *APOBEC3A* (A3A−/GRHL3−); GRHL3 ‘on’, no detectable *APOBEC3A* (A3A−/GRHL3+); GRHL3 ‘off’, *APOBEC3A* expressed (A3A+/GRHL−) and GRHL3 ‘on’, *APOBEC3A* expressed (A3A+/GRHL3+) (bottom). **d)** Histograms showing qRT-PCR-based expression measurements of *APOBEC3A*, *GRHL3, IVL* and *ELF3* in NIKS transfected with control (NC#1) or *GRHL3*-specific siRNAs as indicated. Cells were treated with 100 nM afatinib for 24 hours prior to harvesting to induce differentiation. **(e)** Percentage of DDOST transcripts that were C>U edited at c558 in in NIKS transfected with control (NC#1) or *GRHL3*-specific siRNAs as indicated. Gene expression (d) and DDOST editing (e) in *GRHL3* siRNA-transfected cells was compared with control siRNA-transfected cells using one-way ANOVA with Dunnett’s multiple comparisons test (N = 3, error bars represent SEM; **** = p-value < 0.0001, ***= p-value < 0.001 and ** = p-value < 0.01). **f)** Main panel (top): the regulatory region upstream of *APOBEC3A* visualised on the UCSC genome browser, displaying ChIP-seq binding peaks for GRHL3 and WDR5 in differentiating normal human epidermal keratinocytes (NHEK), GeneHancer regulatory element predictions (grey = enhancer, red = promoter), H3K27Ac, H3K4Me1 and H3K4Me3 ChIP-seq peaks from ENCODE. The GRHL3 binding peak in the −33kb enhancer is highlighted in red. The GRHL3 ChIP-seq trace from proliferating NHEKs^66^, is also shown. Inset (bottom): sequence of the 176 bp GRHL3 binding peak with 8-mers displaying at least 6 matches to the AACCTGTT consensus GRHL3 binding motif shown in red (sense strand) and orange (antisense strand). The logo plot shows the extent in variation of the consensus binding motif across genome-wide GRHL3 binding peaks identified using ChIP-seq data from differentiating NHEKs^65^.

Transcription factor activity analysis of of scRNA-seq data from normal tonsil epithelial cells using single-cell regulatory network inference and clustering (SCENIC)^62^ identified the Grainyhead-like transcription factor 3 (GRHL3), a key regulator of epidermal differentiation^63–66^ as a potential regulator of *APOBEC3A* expression in the datasets from normal tonsil, HNSCC and ESCC, with strong positive associations between GRHL3 activity scores and *APOBEC3A* expression evident across all scRNA-seq datasets we analysed (Table S5). Furthermore, GRHL3 was the only transcription factor among those whose activity was correlated with *APOBEC3A* expression that was significantly upregulated in the differentiating compartment of the normal tonsil epithelium, in which most APOBEC3A-expressing cells were clustered (Figure 4a, b, Figure 1e, Table S6) and it is known to be activated downstream of the Receptor-Interacting Protein Kinase 4 (RIPK4) in PMA-treated keratinocytes^67^. Stratifying cells by their binary (on / off) GRHL3 activity as determined from SCENIC analysis (Figure S3) revealed increased *APOBEC3A* expression in ‘GRHL3-on’ cells (Figure 4c (upper panel); Wilcoxon’s Rank Sum test p<0.0001). 936 of 1,416 (66%) ‘GRHL3-on’ cells expressed *APOBEC3A*, a significantly higher number of cells compared to those that were ‘GRHL3-off’, where only 73 of 1,233 (6%) cells expressed *APOBEC3A* (Fishers Exact Test, p < 0.0001; Figure 4c (lower panel)). GRHL3’s known target genes include *IVL* and E74 Like ETS Transcription Factor 3 (*ELF3*)^65,67^, both of which display very similar patterns of gene expression to *APOBEC3A* in response to differentiation stimuli in NIKS (Figure 3a – c, f Figure S2a, b and Figure S4). Suppressing *GRHL3* expression using two different siRNAs blocked induction of *APOBEC3A* mRNA (Figure 4d) and DDOST mRNA editing (Figure 4e) by afatinib in NIKS, demonstrating a functional role for GRHL3 in activating *APOBEC3A* expression during differentiation. Induction of GRHL3 target genes *IVL* and *ELF3* was also suppressed, whereas expression of *MKI67* and *MCM7* was unaffected by *GHRL3* knockdown (Figure S5), consistent with GHRL3-dependent induction of *APOBEC3A* occurring during afatinib-induced differentiation, downstream of cell cycle exit. Analysis of chromatin immunoprecipitation (ChIP-seq) data from human keratinocytes (NHEK)^65,66^ revealed GRHL3 binding at a predicted enhancer 33kb upstream of the *APOBEC3A* TSS following Ca^2+^-induced differentiation but not in control (proliferating) cells, consistent with a direct role for GRHL3 in regulating *APOBEC3A* transcription (Figure 4f, main panel). This region displays peaks of histone H3K27 acetylation and H3K4 mono-methylation (both marks of enhancers) in cell lines profiled by the ENCODE project (Figure 4f, main panel), and the 176 bp region at which the GRHL3 binding peak is located contains four 8-mer sequences that are close matches for the previously defined consensus GRHL3 binding motif (AACC[G/T]GTT)^64^ (Figure 4f inset). GRHL3 has been shown to recruit the trithorax group (trxG) protein WDR5 to its target sites to enable H3K4 methylation^65^, and a WDR5 binding peak coincided with the GRHL3 peak at −33kb in differentiating NHEKs (Figure 4f, main panel). A second predicted enhancer at −4kb relative to the TSS harbours NFκB and STAT2 binding sites previously implicated in *APOBEC3A* regulation^16,68^, and WDR5 binding was also observed at this region in differentiating NHEKs (Figure 4f, main panel). Together, these data from tissues and cultured cells identify GRHL3 as a key transcription factor that acts to upregulate *APOBEC3A* expression during keratinocyte differentiation.

### GRHL3 drives *APOBEC3A* expression in HNSCC and ESCC

Having determined that *APOBEC3A* is expressed during the terminal differentiation of noncancerous epithelial cells in the tonsil and oesophagus, and that this expression pattern could be recapitulated in immortalised but non-transformed epidermal keratinocytes in culture, we next investigated *APOBEC3A* and *APOBEC3B* expression patterns in scRNA-seq data from tumour samples. Pathway analysis of the top 100 genes co-expressed with either *APOBEC3A* or *APOBEC3B* in 19,314 tumour cells from the 10 Southampton HNSCCs (7 of which were patient-matched with the healthy tonsil samples analysed in Figure 2 (Tables S1, S2)) and in the additional published scRNA-seq datasets from HNSCC^69,70^ and ESCC^71^ revealed similar results to those obtained when performing the analysis on data from healthy tonsil; *APOBEC3A* was again co-expressed with genes in pathways related to keratinocyte differentiation, while *APOBEC3B* was co-expressed with genes in pathways linked to cell division (Figure S6a-d and Tables S7-S10).

**Figure 5:**
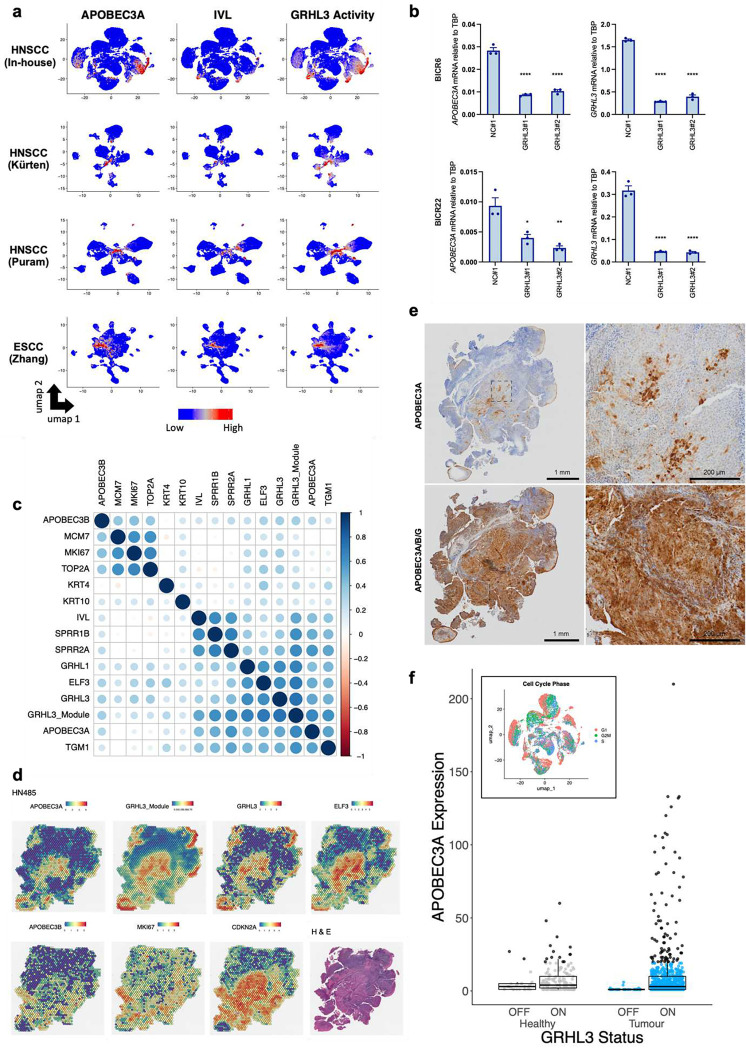
GRHL3 regulates *APOBEC3A* expression in squamous cell carcinoma. **a)** UMAPs heatmap showing gene expression of *APOBEC3A* and *IVL* and predicted activity of GRHL3 in scRNA-seq data from four independent tumour cohorts (3 HNSCC and one ESCC). **b)** Histograms showing qRT-PCR_based expression measurements of *APOBEC3A* and *GRHL3* in BICR6 (top row) and BICR22 (bottom row) HNSCC cells transfected with control (NC#1) or *GRHL3*-specific siRNAs as indicated. Gene expression in *GRHL3* siRNA-transfected cells was compared with control siRNA-transfected cells using one-way ANOVA with Dunnett’s multiple comparisons test (N = 3, error bars represent SEM; **** = p-value < 0.0001; **= p-value < 0.01 and * = p-value < 0.05). **c)** matrix showing the relationship between expression of the indicated genes in spatial transcriptomics data from the Southampton HNSCC cohort obtained using the Visium platform (10X Genomics). **d)** Images displaying expression levels (Visium spot intensities) of selected genes in HN485, an HPV+ve HNSCC case from the Southampton cohort. **e)** immunohistochemistry with an antibody specific for APOBEC3A (left) and with an antibody that cross-reacts with APOBEC3A, APOBEC3B and APOBEC3G (right) in sections from the same tissue block from HN485 used for the Visium profiling displayed in part d. **f)** Boxplot showing expression of *APOBEC3A* in those cells predicted to be in S-phase in normal tonsil and HNSCC, stratified by binary GRHL3 activity score (on/off). Cells shown in black are outliers relative to the distribution of expression in the cells from healthy tonsil. Inset: UMAP showing the predicted cell cycle phase for each cell in the Southampton HNSCC scRNA-seq dataset.

Although it was not possible to visualise the four phenotypes (basal, proliferating, differentiating and terminally differentiated) on UMAPs due to the cells from individual tumours clustering by patient rather than by phenotype (Figure S6e), we again observed *APOBEC3A* co-expression with markers of differentiation and components of the RIPK4 pathway and *APOBEC3B* co-expression with markers of proliferation (Figure S7a). When analysing each of the 10 tumour samples in the Southampton HNSCC dataset individually, the same trends were observed in almost all cases (Figure S7b-k). SCENIC analysis of the four SCC datasets implicated GRHL3 as a potential regulator of *APOBEC3A* in squamous cell carcinoma as well as in healthy epithelia, with strong correlations between GRHL3 activity and *APOBEC3A* expression evident across all studies (Table S5, Figure 5a). *APOBEC3A* expression was also correlated with expression of *GRHL3* and related pathway genes in RNA-seq data from HNSCC cell lines in the Cancer Cell Line Encyclopaedia (CCLE)^72^ (Figure S8a). Among those cell lines profiled by the CCLE, *APOBEC3A* and *GRHL3* mRNA levels were highest in BICR6 and BICR22; lines derived from an SCC of the hypopharynx and from a lymph node metastasis from a tongue SCC respectively^73^ (Figure S8b). *APOBEC3A* and *GRHL3* mRNA levels were higher in sub-confluent cultures of both BICR6 and BICR22 than in NIKS harvested under the same conditions (Figure S9) and we observed a significant reduction in *APOBEC3A* expression upon *GRHL3* knockdown in both cell lines (Figure 5b).

To gain further insight into the heterogeneity of *APOBEC3A* and *APOBEC3B* expression in HNSCC, we next analysed spatial transcriptomics data obtained from tissue sections representing the same cases as those from which our scRNA-seq were derived. Consistent with what we observed in the scRNA-seq analysis, *APOBEC3A* was expressed in regions that displayed high predicted GRHL3 activity (the GRHL3 target genes that comprise the GRHL3 module are listed in Table S11) and expression of additional genes related to keratinocyte differentiation, while *APOBEC3B* was expressed in regions marked by high expression of proliferation markers (Figure 5c). Pathway analysis of genes co-expressed with *APOBEC3A* or *APOBEC3B* yielded similar results to those obtained from the scRNA-seq data but in addition to pathways associated with keratinocyte differentiation, the wound healing response (another process in which GRHL3 plays a critical role) was also overrepresented among those genes co-expressed with *APOBEC3A* (Table S12, Figure S10). Since the Visium platform typically provides resolution of approximately 10 cells / spot depending on cell size and cellularity, we performed spot deconvolution, observing that the *APOBEC3A* reads from each spot were largely derived from epithelial (tumour) cells with expression also evident in monocytes and neutrophils, consistent with previous reports^5,74,75^. *APOBEC3B* reads were largely derived from the tumour cells (Figure S11). A representative example tumour section (case HN485), displaying regions of *APOBEC3A* expression with high GRHL3 activity (‘GRHL3 module’, composed of SCENIC-predicted target genes including *ELF3*) is shown in Figure 5d. In the same section, distinct *MKI-67*-positive regions of the tumour show peak expression of *APOBEC3B*. Strong *CDKN2A* expression (the gene encoding p16^INK4A^, a biomarker for HPV-positive HNSCC) is evident throughout most of the tumour cells. GRHL3 activity and *APOBEC3A* expression were frequently highest near to the tumour surface.

Analysis of APOBEC3 protein expression in tissue samples has been hampered by a lack of suitable antibodies for detection by immunohistochemistry but we (M.A.C and R.S.H) recently developed a monoclonal antibody that specifically detects APOBEC3A in formalin-fixed, paraffin-embedded tissues^22^. Having confirmed specificity by staining of paraffin-embedded blocks generated from PMA-treated wild-type control and *APOBEC3A*-knockout (KO) NIKS (Figure S12a), we conducted APOBEC3A immunohistochemistry on a tissue microarray (TMA) representing 20 HNSCC cases (10 HPV+ve and 10 HPV-ve). As predicted from our scRNA-seq and spatial transcriptomics data, some tumours were devoid of APOBEC3A, while others displayed abundant staining in more differentiated tumour cells, including in those cells surrounding keratin pearls – a distinguishing feature of well differentiated SCC (Figure S12b(I), left panel). Staining the same TMA with an antibody that binds to APOBEC3A, APOBEC3B and APOBE3G^76^ revealed characteristic nuclear APOBEC3B expression in tumour cells (Figure S12b, right panels). As expected, this was particularly evident in HPV-positive cases (Figure S12b (II, III), right panels), in which APOBEC3B is upregulated by the viral E6 and E7 proteins^7,53,54,77^. APOBEC3G is known to be expressed in the cytoplasm of T-lymphocytes and was evident in resident lymphocytes (e.g. Figure S12b(I) arrowheads). The pan-cellular staining of keratinizing cells with the APOBEC3A/B/G antibody is consistent with the APOBEC3A-specific staining (compare Figure S12b(I) boxed areas and S12b(III) insets between left and right panels). We also stained three cases (HN485, Figure 5e), HN482 and HN494 (Figure S13), for which we had also generated spatial transcriptomics data, observing good concordance between the patterns of mRNA and protein positivity for both *APOBEC3A* and *APOBEC3B* (compare Figure 5d and e and Figure S13a and b). Importantly, in addition to providing further validation of the specificity of our antibodies, these data confirm that our conclusions relating to *APOBEC3A* and *APOBEC3B* expression drawn from mRNA data (scRNA-seq, spatial transcriptomics) are valid at the protein level.

Finally, while *APOBEC3A* expression was largely confined to IVL^+ve^ / MKI67^−ve^ (non-cycling) tumour cells, the correlation between *APOBEC3A* and *IVL* expression was weaker in the SCC datasets than in the normal tonsil epithelial cells, and in the UMAPs from the Southampton HNSCC dataset, *APOBEC3A* expression was apparent in IVL^−ve^ cells, which also displayed high predicted GRHL3 activity (Figure 5a). This was most obvious in two tumours (HN489 and HN492; compare Figure 5a and Figure S6e), suggesting that under certain conditions, activation of GRHL3 may induce *APOBEC3A* in cycling tumour cells. Given the considerable evidence linking APOBEC3A-mediated mutagenesis to deamination of the lagging strand during DNA replication^39–44^, we used gene expression data to assign cells from our normal tonsil and HNSCC datasets to G0/G1, S, or G2/M phase of the cell cycle (Figure 5f inset) and compared *APOBEC3A* expression and GRHL3 activity in those cells predicted to be in S-phase. While as expected, the majority of S-phase cells did not express *APOBEC3A*, considering all S-phase cells in which *APOBEC3A* expression was detectable (more than zero reads) we observed a small minority that expressed considerably more *APOBEC3A* mRNA than was seen in S-phase cells from normal tonsil epithelium (Figure 5f; black dots on the boxplot in represent cells that are statistical outliers with respect to the level of *APOBEC3A* found in normal tonsil). The S-phase tumour cells with high *APOBEC3A* expression were all designated a binary GRHL3 activity score of ‘on’, suggesting that GRHL3 can drive *APOBEC3A* expression in tumour cells undergoing DNA replication, potentially causing APOBEC3A-mediated mutagenesis. The fact that we only observed high *APOBEC3A* expression in a small minority of S-phase cells in our HNSCC samples is consistent with the proposed episodic nature of APOBEC-mediated mutagenesis, in which the chances of observing a mutagenic burst in the snapshot provided by a tumour biopsy are low^31,32,78,79^.

## Discussion

Our analysis of *APOBEC3A* and *APOBEC3B* gene expression in healthy and cancerous squamous epithelia provides new insight into how these genes are regulated and raises several questions that warrant further investigation. The low expression of *APOBEC3B* in normal epithelium and increased levels in tumours that we observed in scRNA-seq datasets is consistent with previous analyses of bulk tissue samples and breast cancer cell lines^26^, in which repressive E2F/RB complexes have been shown to silence expression in quiescent cells^38,54,80^. Loss of p53-mediated repression of *APOBEC3B* transcription, resulting either from *TP53* mutation (observed at high frequency in HPV-negative HNSCC and ESCC) or from HPV E6/E7 activity in HPV-positive HNSCC^53,54^ is also likely an important driver of *APOBEC3B* expression seen in many of the SCC samples we analysed.

The high expression of *APOBEC3A* in tonsil and oesophageal epithelium could indicate a role in defence against one or more viruses with tropism for the upper aerodigestive tract. Wild-type adeno-associated virus (AAV, a target for APOBEC3A^5^) infects keratinocytes via binding to heparan sulphate proteoglycans and AAV genomic DNA has been isolated from tonsils^81^. *APOBEC3A* and *APOBEC3B* have both been implicated in host responses to HPV infection^6,7,53,82^ and although our data suggest that neither are expressed in quiescent basal cells of the tonsil epithelium (the target cell for HPV infection), *APOBEC3B* is expressed in those cells undergoing division in the parabasal layer, while *APOBEC3A* is expressed in cells undergoing terminal differentiation; a pattern also evident in an area of normal stratified epithelium at the margin of an HPV-associated oropharyngeal SCC in which APOBEC3A and APOBEC3B were detected by RNA *in situ* hybridization^83^. Both genes are therefore expressed (at least in the absence of infection) under cellular conditions in which different stages of the HPV productive life cycle occur: genome maintenance following E6/E7-induced cell cycle entry in the basal / para-basal layer and genome amplification in terminally differentiating cells^84^. Whether this pattern of APOBEC3 gene expression represents a host adaptation to papillomaviruses, or to other pathogens that infect the upper-aerodigestive tract remains to be determined. Alternatively, the expression of *APOBEC3B* in dividing keratinocytes and *APOBEC3A* during terminal differentiation may reflect hitherto unidentified physiological roles related to these processes. *APOBEC3B* is also expressed as breast cancer cell lines approach mitosis and its knockdown slows proliferation, suggesting a role in cell cycle progression that might be linked to its function as a transcriptional co-activator for the oestrogen receptor^3,38^. Unlike in normal breast epithelium (Figure 1) or in MCF10A, a cell line derived from normal mammary epithelium^38^, we observed sufficient *APOBEC3B* expression in our scRNA-seq data from normal tonsil epithelium and from NIKS (Figure 3e) to observe a clear enrichment in G2/M-phase cells, suggesting APOBEC3B may play a role in normal keratinocytes entering cell division. *APOBEC3B* expression in G2/M-phase is not unique to epithelial cells either; it has also been documented in myeloma cells and in B-cells from healthy bone marrow^37^. *APOBEC3A* induction during Ca^2+^induced keratinocyte differentiation has been linked to hypermutation of mitochondrial DNA^52^, although the functional significance of this remains unclear. More investigation of *APOBEC3A* and *APOBEC3B* function in epithelial cells is required but it is maybe not surprising that by restricting *APOBEC3A* expression to post-mitotic keratinocytes and *APOBEC3B* expression to the G2/M phase of proliferating cells, mechanisms have evolved to restrict these potentially dangerous deaminases to contexts in which DNA replication is not occurring.

The identification of GRHL3 as a transcription factor responsible for driving *APOBEC3A* expression in differentiating keratinocytes and in squamous cell carcinoma highlights the power of single cell transcriptomics to uncover gene regulatory networks. In this case using SCENIC^62^ we observed striking correlations between GRHL3 activity scores and *APOBEC3A* expression across multiple scRNA-seq datasets from healthy and cancerous epithelia and validated the prediction using RNA interference in cultured cells. GRHL3 is a key transcription factor in epidermal keratinocytes, required not only during differentiation but also in migration during developmental processes and in wound healing^63,64,85–88^. Given the extensive overlap between the molecular processes that are active during wound healing and cancer^89^, this latter function may be of particular relevance to driving *APOBEC3A* expression in tumour cells, including in those undergoing DNA replication and warrants further investigation.

While most studies have focused on its function in the epidermis, *GRHL3* and its murine orthologue, *Grhl3*, have been implicated as suppressors of squamous carcinogenesis in the mucosal epithelia of the oral cavity and oesophagus as well as in skin^90,91^. *GRHL3* loss-of-function mutations have not been reported in SCC but it is located at a locus (1p36.11) that is frequently deleted in HNSCC and it is also targeted by a micro-RNA (miR-21) that is over-expressed in HNSCC. Accordingly, *GRHL3* expression in SCCs was demonstrated to be significantly lower than in adjacent normal tissue^90,92^. Our scRNA-seq analysis of normal tonsil and HNSCC cases is agreement with the above studies; we observed higher mean *APOBEC3A* expression in normal tonsil epithelial cells than in tumour cells from patient-matched HNSCC cases (Figure 1). Similarly, while spatial transcriptomics and immunohistochemistry of sections from these HNSCCs revealed widespread *APOBEC3B* expression (particularly in HPV +ve cases, as expected), *APOBEC3A* was not expressed in all cases and in those tumours where expression was observed, it was restricted to areas of high GRHL3 activity (Figure 5).

These observations, together with our siRNA experiments in HNSCC cell lines, suggest that in SCC at least, *APOBEC3A* expression is confined to the minority of tumour cells in which *GRHL3* is expressed and active. If we consider that APOBEC activity is only likely to be mutagenic in cycling cells (or in cells that have re-entered the cell cycle without repairing deaminated cytosines), the pool of tumour cells at risk of APOBEC3A-mediated mutagenesis is likely limited to those rare cells highlighted in Figure 5f, in which *APOBEC3A* expression coincides with DNA replication. It follows that if such *APOBEC3A*-expressing tumour cells were to acquire mutations that caused them to become more proliferative (as might be expected if the subclone were to expand to constitute a significant portion of the tumour) this would result in loss of *APOBEC3A* expression (and potentially an increase in *APOBEC3B* expression). This model could explain the somewhat puzzling observation that tumours with strong enrichment for mutational signatures (YpTp[C>T/C>G]pN) associated most strongly with APOBEC3A often express very low levels of *APOBEC3A*, while *APOBEC3B* expression is more closely correlated with enrichment for the APOBEC mutational signatures but appears to be responsible for generating a smaller fraction of these mutations^30,31,93,94^.

Finally, we note that activation of GRHL3 in keratinocytes plays a key role in resolving psoriatic lesions by suppressing inflammatory mediators and driving epidermal repair^87,95^. Our finding that GRHL3 regulates *APOBEC3A*, which was originally discovered as a protein (Phorbolin-1) that is highly upregulated in psoriatic lesions^55,56^ finally provides a potential mechanistic explanation for this early observation that predated mapping of the *APOBEC3A* gene^96^ by almost a decade.

## Methods

### Ethics / patient samples

Patients undergoing biopsies of suspected primary Head and Neck cancers at University Hospitals Dorset (UHD) NHS Foundation Trust were consented to take part in a study; “Head and Neck cancer: molecular, cellular and immunological mechanisms”. This study is NIHR portfolio adopted (portfolio No. 8130) and has been approved by the National research ethics service South Central committee (reference No. 09/H0501/90). Tumour samples from ten oropharyngeal patients (Supplementary Table 1), as well as normal tissue samples from the contralateral tonsil for seven of the patients (collected at the time of diagnostic biopsy) were selected for single cell RNA sequencing.

### Single cell suspension preparation

Upon receipt, tissue samples were washed once in Dulbecco’s modified eagle medium (Sigma #D5671) containing 10% Foetal Calf Serum, 1% Penicillin/streptomycin, 1% L-Glutamine, 1% Amphotericin, 1% Sodium pyruvate, and 12.5mM HEPES. Samples were chopped into 1–2mm size pieces prior to enzymatic digestion. The first stage of the enzymatic digestion was performed using Liberase^™^ (Sigma #5401020001) at 100μg.mL^−1^ and DNase-1 (Sigma #DN25) at 16 units.mL^−1^ in cDMEM. The solution was sterile filtered using a 0.22μm syringe filter and the sample material was suspended in up to 5mL of cDMEM/Liberase solution. Samples were then sealed and placed in a benchtop shaker/incubator at 37°C and 150rpm for fifteen minutes and then removed. The tube was left to stand until the undigested material had settled to the bottom then the upper 4 – 4.5mL was carefully transferred to a fresh tube, the Liberase fraction. For the second digest (Col+) cDMEM containing collagenase-P (Sigma #11213857001) at 3 units.ml^−1^, liberase at 100μg.mL^−1^, dispase (Sigma #D4693) at 0.5 units.mL^−1^, elastase (Sigma #E1250) at 400μg.mL^−1^, trypsin (Sigma #T4799) at a final concentration of 0.25%, and DNase-1 (16 units.mL^−1^) was added to the remaining material through a 0. 22μm sterile syringe filter. The Col+ digest was returned to the incubator (37°C / 150rpm) for up to a maximum of 45 minutes (or until digestion is complete) with trituration performed using a 5mL graduated pipette every 15 minutes. After 45 minutes the Col+ fraction was removed from the incubator and any remaining undigested pieces were allowed to settle at the bottom of the tube; the supernatant was then transferred to a fresh sterile tube. Any remaining tissue was set aside.

The post digestion process was the same for both the Liberase and Col+ fractions. Complete DMEM, up to 10 mL, was added to each fraction and both cell suspensions were pelleted at 350rcf for 5 minutes. Supernatant was removed and RBC lysis buffer (Biolegend #420301) used to remove erythrocytes for 10 minutes at 4°C. The samples were then washed in PBS and suspended in residual volume and then held at 4°C until the Col+ fraction was prepared. Cell pellets were suspended in PBS containing 2% BSA-Fraction V (Scientific Lab Supplies #10735108001) and passed through a pre-wetted 40μm filter. Both samples were then counted and viability assessed by Trypan blue exclusion. A final visual check of sample quality was also performed to ensure there were no large clumps of cells nor debris from the digestion. Finally the two fractions were used to make a 100μL suspension of 100,000 cells of which 10,000 were from the liberase fraction and 90,000 from the Col+ fraction, and 2% BSA in PBS was used as the diluent. This cell suspension was then run immediately on a Chromium Controller (10X Genomics).

### Fluorescence-activated cell sorting

Flow cytometry to determine the proportions of cell types in the disaggregated samples was carried out using a FACSCanto II (BD Biosciences). Cell viability was assessed using Zombie Violet^™^ (Biolegend #423114). The following antibodies were purchased from Biolegend: EpCAM (#369806), CD90 (#328114), CD45 (#368508), CD31 (#303118), CD3 (#300426). A minimum of 20,000 events were acquired for each case. Gating was applied to exclude debris, dead cells, doublets and the immune compartment (CD45+ and/or CD3+) before enumerating the numbers of endothelial, epithelial, and CD90 positive fibroblasts in the sample.

### Single cell RNA sequencing

Five thousand single cells from each sample were captured on a Chromium Controller^™^ (10X Genomics) system using Illumina single cell 3’ gene expression and library preparation kits (V3.1 #1000269). Sample capture, sample indexing, and library preparation were carried out strictly according to manufacturer’s instructions. Size distribution, quality control, and quantification of the libraries was assessed using High Sensitivity DNA chips (Agilent Technologies #5067–4626) and KAPA library quantification qPCR kit (Roche #07960140001). Prepared libraries were pooled and sent to Oxford Genomics (UK) for 150-base pair, paired-end sequencing on a Novaseq6000^™^.

### Sequence alignment and annotation

Cell Ranger (10x Genomics) pipelines (mkfastq, count) were used to align reads, filter, count barcodes and UMIs (unique molecular identifiers) and generate feature-barcode matrices. FASTQ files were aligned to the Human reference genome (GRCh38–2020-A) which had the HPV genome concatenated to both FASTA and GTF reference files. HPV reference sequences were downloaded from PaVE: The Papillomavirus Episteme (https://pave.niaid.nih.gov). The HPV-16 reference sequence (NC_001526) was used in the first instance and in cases requiring further identification of the HPV subtype references including HPV-33 (OQ_672679) and HPV-18 (NC_001357) were also created. In all cases the individual HPV ORFs were identified in the FASTA and GTF files to allow identification during alignment.

### Pre-processing of scRNA-seq data

For each sample, raw gene expression matrices were integrated into one dataset using Seurat package (v4.0.1). The resulting feature-barcode matrix from the cell ranger pipeline was transformed into a Seurat object with patient metadata. Cells with less than 200 expressed genes were removed. Genes expressed in less than 3 cells were also filtered out. Further low-quality cells were removed based on mitochondrial gene percentage with the threshold for calculated as the median + 3*median absolute deviation. Cells above the threshold were removed ensuring high quality cells remained.

### Normalisation and integration (EPG)

After quality control steps, the data was normalised to adjust for differences in sequencing depth between samples. sctransform was chosen to normalise and variance stabilize the count data^97^. Reciprocal PCA (‘RPCA’) Seurat integration workflows were utilised for integration. The Seurat object was first split by patient into a list of 10 smaller objects, in which each dataset was normalised by sctransfrom individually. 3000 features were selected via ‘SelectIntegrationFeatures’ function. ‘PrepSCTIntegration’ was run prior to anchor identification to ensure sctransfrom residuals from the 3000 features identified (by SelectIntegrationFeatures) were present. Anchors, used to integrate objects, were found between datasets using FindIntegrationAnchors, with the normalization.method set to ‘SCT’ and reduction set to ‘rpca’ (all other parameters were default). Finally, IntegrateData was run, again specifying ‘SCT’ as normalization.method. This integration pipeline was run using IRIDIS High Performance Computing Facility (University of Southampton).

### Dimensionality reduction, visualisation, and clustering

Principal component analysis (PCA) was used to reduce the dimensionality of the datasets. Principal components were assessed by JackStraw and elbow plots to select an appropriate number of dimensions to be used downstream. Dimensions 1:30 were selected in the following steps. Clustering was performed in Seurat, which constructs a k-nearest neighbours graph and refines this using the shared local neighbourhood overlap between cells (‘FindNeighbours’; ‘FindClusters’). RunUMAP command was used to visualise the data in a UMAP (Uniform, Manifold, Approximation and Projection) plot.

### Identification of marker genes and cell type identification

After clustering and UMAP projection, broad cell populations were identified based on expression of known marker genes e.g., PTPRC/CD45+ immune cells, LUM+ Fibroblasts, RGS5+ Mural cells, PECAM1/CD31+ endothelial cells. Epithelial cell clusters were identified based on the expression of EPCAM, SFN and cytokeratin genes (e.g., KRT14, KRT17, KRT6A, KRT5, KRT19) – with absence of expression of other cell-type markers. Epithelial cell clusters were then subset into a separate object, with new variable features found by re-running sctransform, PCA and clustering, whereby any clusters suggestive of doublets were removed based on the expression of non-epithelial markers (identified using FindAllMarkers) and examining UMI/feature number. The remaining epithelial cells were then used for further analysis.

### Unsupervised clustering of epithelial cells

A total of 22,595 epithelial cells were subset into tumour (19,314 cells) and normal (3,281 cells) Seurat objects. The tumour cells were clustered using the first 15 principal components with a resolution of 0.2 and k parameter of 60. The normal epithelial cells underwent further quality control, 658 cells were removed as suspected doublets due to high expression of immune cell and fibroblast related genes. The remaining 2,623 cells were clustered using the first seven principal components with a resolution of 0.2, and a k parameter of 30. Cell subtypes were identified using known gene markers for epithelial and keratinocyte cell states, and *a priori* knowledge. The density of APOBEC3A and APOBEC3B expression was visualised on UMAPs using the Nebulosa R package (v1.9.0).

### Gene co-expression

The COTAN R package (v2.0.1) was used to investigate the co-expression of gene pairs for scRNA-seq datasets. For both *APOBEC3A* and *APOBEC3B*, the top 100 genes with a positive correlation index were identified and used in pathway analysis using the enrichR package (v3.2) and GO biological processes gene sets. Relevant epithelial differentiation and proliferation markers were chosen and *APOBEC3A*/*APOBEC3B* co-expression values with selected genes were plot in heatmaps using R package pheatmap (v.1.0.12).

### SCENIC analysis

Transcription factor (TF) analysis of scRNA-seq data was performed using pySCENIC (v0.12.1) [DOI: 10.1007/978-1-0716-1534-8_10] and motif collection version mc9nr. TF activity AUC score for each cell was overlaid on UMAPs for visualisation and the score was correlated with *APOBEC3A* and *APOBEC3B* expression using Spearman correlation and corrected for multiple tests using Benjamini-Hochberg. Binary activity (on/off) of each TF was determined based on a threshold generated by pySCENIC and each cell was classified as on (1) or off (0). The *APOBEC3A* levels in ‘GRHL3 on’ and ‘GRHL3 off’ cells were compared using Wilcoxon rank sum test. Four groups of *APOBEC3A*/*GRHL3* expression were considered: *APOBEC3A*^−^/*GRHL3*^−^, *APOBEC3A*^+^/*GRHL3*^−^, *APOBEC3A*^−^/*GRHL3*^+^, and *APOBEC3A*^+^/*GRHL3*^+^. The number of cells in each group were counted and a comparison between the number of ‘*GRHL3* on’ (*GRHL3*^+^) cells that were also *APOBEC3A*^+^ were compared with the number of ‘*GRHL3* off’ (*GRHL3*^−^) cells that were *APOBEC3A*^+^ using Fisher’s exact test. The FindAllMarkers function in Seurat R package was used to perform the differential transcription factor activity analysis, using a threshold fold change of 1.1 and Benjamini-Hochberg adjusted p-value of 0.05.

### GHRL3 binding motif analysis

Homer (v4.11) function findMotifsGenome with mismatches threshold set to 2 was used to identify the frequency of GRHL3 binding motifs in differentiating NHEK ChIP-seq for GRHL3 peaks. The three binding motifs searched for were based on previous literature: AACCGGTT^98^, AACCTGTT and AACAGGTT^64^. The percentage of times each base was located at each position in the binding motif was calculated and visualised using R package motifStack (v1.44.1) and dependencies.

### Spatial transcriptomic analysis

All steps leading up to sequencing (from the bench side) were performed per manufacturer recommendations on 6.5mm capture areas using the Visium V2 cytassist workflow. All the samples were run through the Spaceranger pipeline (v2.0.0) as per 10X Genomics/Visium guidelines.

Count matrices were loaded into Seurat. Samples were normalised using SCTransform function (using the variance-stabilizing transformation). To identify spot clusters across patients, samples were integrated using the Seurat v3 CCA anchor finding method (FindIntegrationAnchors and IntegrateData). The 3000 variable features selected for integration were then used for principal component analysis (PCA), followed by FindNeighbors and FindClusters for (shared) nearest-neighbor graph construction and cluster determination respectively. Uniform manifold approximation and projection (UMAP) algorithm (1:20 dimensions) was used to visualise the batch corrected integrated dataset. Resulting clusters were inspected, with poor quality clusters removed. The GRHL3 module score for each spot was calculated using AddModuleScore with 127 genes identified by SCENIC as potential target genes for GRHL3 binding in normal epithelium and HNSCC. Spatial feature expression plots were generated with the SpatialFeaturePlot function in Seurat.

Robust Cell Type Decomposition (RCTD) with spacexr 2.2.1^99^ in R was used to deconvolve Visium spots into cell types using the annotated scRNA-Seq HNSCC reference dataset. RCTD was ran with default parameters and doublet mode set to ‘full’ on each individual patient sample, with resulting deconvoluted normalised weights for each cell type obtained.

### APOBEC3A expression in cancer cell lines

Expression data for 34 head and neck cancer cell lines from the Cancer Cell Line Encyclopaedia (CCLE)^100^ was obtained from the resource CellminerCDB^101^ for differentiation and proliferation marker genes, *APOBEC3A*, and genes in the RIPK4 pathway. Spearman correlation coefficients were calculated pairwise for all genes.

### Tissue microarrays

Tissue microarrays (TMAs) were constructed from paraffin-embedded HNSCC and normal oral mucosa (10 HPV+ve HNSCC, 10 HPV-ve HNSCC, 10 fibroepithelial polyps) using triplicate, randomly selected, 1-mm tumour cores (Aphelys Minicore 2, Mitogen, Harpenden, UK). Automated immunostaining (DAKO/Agilent Autostainer) was performed in a CPA-accredited clinical cellular pathology department.

### Immunohistochemistry

Staining of tissue microarrays and full-face sections was performed on a Dako link automated staining machine according to the manufacturer’s instructions. The following antibodies were used: rabbit monoclonal anti-human APOBEC3A/B/G (Cell Signaling Technology, Cat#81001; 1:100 dilution with Dako FLEX TRS high pH retrieval); rabbit monoclonal anti-human APOBEC3A (UMN-13^22^); 1:1000 dilution with Dako FLEX TRS high pH retrieval).

### Cell culture

Low-passage Normal immortalised keratinocytes (NIKS) were cultured in FC medium (3:1 Ham’s F12:DMEM, 5% foetal bovine serum, 10 ng/ml murine submaxillary gland EGF, 24 μg/ml adenine, 5 μg/ml insulin, 8.3 ng/ml cholera toxin, 0.4 μg/ml hydrocortisone, 1% penicillin/streptomycin) on a feeder layer of mitomycin C-treated mouse embryonic fibroblasts (J2–3T3). BICR6 and BICR22 cells were cultured in DMEM supplemented with 10% foetal bovine serum, 2 mM L-Glutamine, 0.4 μg/ml hydrocortisone and 1% penicillin/streptomycin. Cells were routinely checked and confirmed mycoplasma-negative by qPCR (Mycoplasmacheck, Eurofins Genomics) upon thawing and were subsequently used for experiments within 2–3 passages.

### qRT-PCR

RNA purification was performed using the Monarch Total RNA Miniprep Kit (NewEngland BioLabs) and on-column DNase digestion. cDNA was synthesised from RNA using LunaScript Reverse Transcriptase (RT) SuperMix Kit (NewEngland BioLabs). Gene-specific primers were synthesised by IDT and are shown in Table 1. The qRT-PCR primers for *APOBEC3A*, *APOBEC3B* and TATA binding protein (*TBP*) were published previously^102^ and the remaining qRT-PCR primers were designed using OriGene’s qPCR primer design tool (https://www.origene.com). All real-time PCR reactions were performed using duplicate technical repeats on a QuantStudio Real-Time PCR system (Applied Biosystems) with amplification using SYBR Green PCR Master Mix (Applied Biosystems). The thermal cycling conditions were at 50°C for 2 min followed by an initial denaturation step at 95°C for 10 min, 40 cycles at 95°C for 15s and 60°C for 1 min, followed by 95°C for 1 min, 60°C for 1 min and 95°C for 1s. Standard curves for *APOBEC3A* and *APOBEC3B* were derived using plasmids pRH3097-A3A (R.S.H lab) and pCMV4-APOBEC3B (a kind gift from Prof Mike Malim, Kings College London, UK) respectively. Standards for all other qRT-PCR target genes were constructed by cloning PCR amplicons generated from a NIKS cDNA library into pCR^™^ Blunt II-TOPO^™^ using the Zero Blunt^™^ TOPO^™^ PCR cloning kit (Thermo Fisher) as per the manufacturer’s instructions. PCR was conducted using the KAPA HiFi 2X MasterMix (Roche) according to the manufacturer’s instructions with 10 ng input cDNA and for all target genes except *TBP* (for which additional primers are listed in Table 1), amplicons were generated using the qRT-PCR primers. All plasmids are available from the authors upon request.

### siRNA transfections

Silencer Select small interfering RNAs (siRNA) were purchased from ThermoFisher Scientific (Negative control (NC#1) Cat No 4390843; GRHL3 (#1) Cat No s33753; GRHL3 (#2) Cat No s33754). NIKS were plated in 6-well plates at a density of 2 × 10^5^ cells / well and BICR6, BICR22 at 1.5 × 10^5^ cells / well, with 1 × 10^5^ feeder cells / well and were transfected with 2 nM of siRNA using 2 uL of Lipofectamine RNAiMAX (ThermoFisher Scientific) per well according to the manufacturer’s instructions (reverse transfection method). Transfection complexes were removed after 24h, followed by (NIKS) 48h recovery period and 24h treatment with 100 nM afatinib to induce differentiation, or (BICR6, BICR22) 42h recovery period with a media change 18hr prior to cell collection.

### DDOST RNA editing assay

DDOST editing at C558 was measured as described previously^60^, using 125ng input RNA for cDNA synthesis. Digital PCR was conducted using the Absolute-Q instrument (ThermoFisher), with 1 uL of 1:4-diluted cDNA.

## Figures and Tables

**Table 1:** Primers used for qPCR and/or plasmid construction.

Gene	Sequence

APOBEC3A (Forward)	5’-GAGAAGGGACAAGCACATGG-3’
APOBEC3A (Reverse)	5’-TGGATCCATCAAGTGTCTGG-3’
APOBEC3B (Forward)	5’-GACCCTTTGGTCCTTCGAC-3’
APOBEC3B (Reverse)	5’-GCACAGCCCCAGGAGAAG-3’
KRT10 (Forward)	5’-CCTGCTTCAGATCGACAATGCC-3’
KRT10 (Reverse)	5’-ATCTCCAGGTCAGCCTTGGTCA-3’
IVL (Forward)	5’-GGTCCAAGACATTCAACCAGCC-3’
IVL (Reverse)	5’-TCTGGACACTGCGGGTGGTTAT-3’
MKI67 (Forward)	5’-GAAAGAGTGGCAACCTGCCTTC-3’
MKI67 (Reverse)	5’-GCACCAAGTTTTACTACATCTGCC-3’
MCM7 (Forward)	5’-GCCAAGTCTCAGCTCCTGTCAT-3’
MCM7 (Reverse)	5’-CCTCTAAGGTCAGTTCTCCACTC-3’
ELF3 (Forward)	5’-CATGACCTACGAGAAGCTGAGC-3’
ELF3 (Reverse)	5’-GACTCTGGAGAACCTCTTCCTC-3’
GRHL3 (Forward)	5’-ACTGTGGAGCACATTGAGGAGG-3’
GRHL3 (Reverse)	5’-CTGTGCTCAGACAGTTTACGCC-3’
TBP (Forward)	5’-TTGAGGAAGTTGCTGAGAAGAG-3’
TBP (Reverse)	5’-CAGATAGCAGCACGGTATGAG-3’
TBP standard (Forward)	5’-CACTCACAGACTCTCACAACTG-3’
TBP standard (Reverse)	5’-GTCGTCTTCCTGAATCCCTTTAG-3’

## Data Availability

Our scRNA-seq data will be made available upon publication. Validation/external scRNA-seq data-sets are available for healthy lung and oesophagus^50^ at Human Cell Atlas Data Coordination Platform and NCBI BIOPROJECT accession code PRJEB31843, and the following at the Gene Expression Omnibus (GEO): breast (GSE176078)^103^; healthy skin (GSE130973)^104^; bladder squamous cell carcinoma (GSE190888)^105^; head and neck squamous cell carcinoma (GSE164690^70^ and GSE182227^69^); lung carcinoma (GSE131907, GSE136246, GSE148071, GSE153935, GSE127465, GSE119911)^106^, collected at https://doi.org/10.6084/m9.figshare.c.6222221.v3; oesophageal squamous cell carcinoma (GSE160269)^71^. Cancer cell line data were obtained at https://discover.nci.nih.gov/rsconnect/cellminercdb/ (accessed 31/07/2023).
